# P-299. No Clinically Significant Drug-Drug Interactions Between Lenacapavir and Hormonal Contraceptives in PURPOSE 1

**DOI:** 10.1093/ofid/ofaf695.519

**Published:** 2026-01-11

**Authors:** Disebo Potloane, Cheryl E Louw, Godfrey Kigozi, Moelo Malahleha, William Brumskine, Amy Ward, Dhayendre Moodley, Alexander Kintu, Marjorie Z Imperial, Priyanka Arora, Renu Singh, Lillian B Brown, Christoph C Carter, Flavia Matovu Kiweewa

**Affiliations:** Centre for the AIDS Programme of Research in South Africa CAPRISA, Durban, KwaZulu-Natal, South Africa; Madibeng Centre for Research, Brits, North-West, South Africa; Africa Medical and Behavioral Sciences Organization, Kalisizo, Lwengo, Uganda; Synergy Biomed Research Institute, East London, Eastern Cape, South Africa; The Aurum Institute, Rustenburg, North-West, South Africa; Vuka Research Clinic, University of Cape Town, Cape Town, Western Cape, South Africa; Centre for the AIDS Programme of Research in South Africa, University of KwaZulu-Natal, Durban, KwaZulu-Natal, South Africa; Gilead Sciences, Inc., Foster City, California; Gilead Sciences, Inc., Foster City, California; Gilead Sciences, Inc., Foster City, California; Gilead Sciences, Inc., Foster City, California; Gilead Sciences, Inc., Foster City, California; Gilead Sciences, Inc., Foster City, California; Makerere University—Johns Hopkins University Research Collaboration, Kampala, Kampala, Uganda

## Abstract

**Background:**

Twice-yearly subcutaneous (SC) lenacapavir (LEN) demonstrated efficacy and safety for HIV pre-exposure prophylaxis (PrEP) in the Phase 3 randomized PURPOSE 1 (NCT04994509) trial in cisgender women. LEN is a CYP3A inhibitor and thus has the potential to increase concentrations of hormonal contraceptives metabolized by the CY3PA pathway. Therefore, we assessed drug–drug interactions between LEN and commonly used progestin-type long-acting (LA) hormonal contraceptives in PURPOSE 1 participants.

**Figure:**
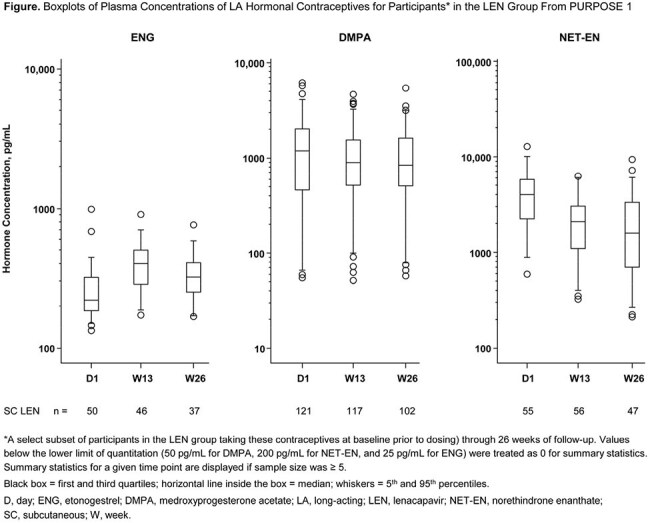

**Methods:**

PURPOSE 1 participants were randomized 2:2:1 to SC LEN or oral F/TAF or F/TDF. Free contraception was provided but not required. We evaluated etonogestrel (ENG), medroxyprogesterone acetate (DMPA), and norethindrone enanthate (NET-EN) concentrations based on observed levels in a selected subset of participants in the LEN group taking these contraceptives at baseline (prior to dosing) through 26 weeks (Ws) of follow-up. Using a population pharmacokinetics (popPK) model, we assessed the impact of hormonal contraceptives on LEN concentrations. Adverse events (AEs) for relevant groups were assessed.

**Results:**

Of 2140 participants receiving LEN, 226 had uninterrupted LA hormonal contraceptive exposure through W26 (50 ENG, 121 DMPA, 55 NET-EN). Plasma concentrations of DMPA and NET-EN at W13 and W26 were comparable with baseline levels, irrespective of dose, frequency, or dosing changes across visits (Figure). Median ENG concentrations showed an upward trend at W13, before decreasing towards baseline levels at W26. Incidence of common ENG-related AEs was similar in the LEN and combined oral PrEP groups (headache, 11% vs 16%; heavy menstrual bleeding, 4% vs 5%). In the popPK analysis, no statistically significant impact of LA hormonal contraceptive use was observed on LEN PK.

**Conclusion:**

LEN coadministration did not result in clinically significant changes in the PK of ENG, DMPA, and NET-EN progestin-type LA hormonal contraceptives, nor did these contraceptives impact LEN PK. Similar findings for LEN were observed with feminizing gender affirming hormonal therapy (Blumenthal et al, abstract submitted to IDWeek 2025). Taken together, these data suggest that no dose adjustments are needed when administering LEN with commonly used LA or oral hormonal contraceptives.

**Disclosures:**

Disebo Potloane, MB, ChB, Dip. HIV Man, Aspire Scientific: Medical writing support|Gilead Sciences, Inc.: Medical writing support Cheryl E. Louw, MB, ChB, Aspire Scientific: Medical writing support|Gilead Sciences, Inc.: Medical writing support Godfrey Kigozi, MB, ChB, Aspire Scientific: Medical writing support|Gilead Sciences, Inc.: Medical writing support Moelo Malahleha, MB, ChB, Aspire Scientific: Medical writing support|Gilead Sciences, Inc.: Medical writing support William Brumskine, MB, ChB, Dip. HIV Man (SA), Aspire Scientific: Medical writing support|Gilead Sciences, Inc.: Grant/Research Support|Gilead Sciences, Inc.: Medical writing support Amy Ward, MBBCh, Dip. HIV Man, Aspire Scientific: Medical writing support|Gilead Sciences, Inc.: Grant/Research Support|Gilead Sciences, Inc.: Medical writing support Dhayendre Moodley, PhD, Aspire Scientific: Medical writing support|Gilead Sciences, Inc.: Medical writing support Alexander Kintu, MB, ChB, ScD, Aspire Scientific: Medical writing support|Gilead Sciences, Inc.: Medical writing support and Employee|Gilead Sciences, Inc.: Stocks/Bonds (Public Company) Marjorie Z. Imperial, PhD, Aspire Scientific: Medical writing support|Gilead Sciences, Inc.: Medical writing support and Employee|Gilead Sciences, Inc.: Stocks/Bonds (Public Company) Priyanka Arora, PhD, Aspire Scientific: Medical writing support|Gilead Sciences, Inc.: Medical writing support and Employee|Gilead Sciences, Inc.: Stocks/Bonds (Public Company) Renu Singh, PhD, MS, Aspire Scientific: Medical writing support|Gilead Sciences, Inc.: 1475-US-PSP, 1475-WO-PCT, 1474-US-PSP, 1515-US-PSP, 1515-WO-PCT|Gilead Sciences, Inc.: Medical writing support and Employee|Gilead Sciences, Inc.: Stocks/Bonds (Public Company) Lillian B. Brown, MD, PhD, Aspire Scientific: Medical writing support|Gilead Sciences, Inc.: Medical writing support and Employee|Gilead Sciences, Inc.: Stocks/Bonds (Public Company) Christoph C. Carter, MD, PhD, Aspire Scientific: Medical writing support|Gilead Sciences, Inc.: Medical writing support and Employee|Gilead Sciences, Inc.: Stocks/Bonds (Public Company) Flavia Matovu Kiweewa, MB, ChB, MSc, PhD, Aspire Scientific: Medical writing support|Gilead Sciences, Inc.: Medical writing support

